# An Index Approach to Early Specialization Measurement: An Exploratory Study

**DOI:** 10.3389/fpsyg.2020.00999

**Published:** 2020-05-26

**Authors:** Charlotte Downing, Karin Redelius, Sanna M. Nordin-Bates

**Affiliations:** Swedish School of Sport and Health Sciences, Stockholm, Sweden

**Keywords:** early specialization, youth sport, developmental pathways, aesthetic sport, dance, measurement

## Abstract

The methodological underpinnings of studies into early specialization have recently been critiqued. Previous researchers have commented on the variety of, and over-simplified, methods used to capture early specialization. This exploratory study, therefore, suggests a new direction for how early specialization can be conceptualized and measured. We aim to create an index approach whereby early specialization is measured as a continuous variable, in line with commonly used definitions. The continuous variable for degrees of early specialization is calculated from a questionnaire which captures the four key components of early specialization; (1) intensity, (2) year-round training, (3) single sport, and (4) commencing age 12 or younger. The proposed index approach is illustrated in a sample of 290 Swedish aesthetic performers aged 12–20 years (*M* = 15.88), whose descriptive statistics are used to discuss the suitability and usability of the measure. The proposed index approach functions as a guideline to future researchers. We hope that introducing a new index approach we will encourage further discussion around the measurement of early specialization. Additionally, we hope to pave the way for future research to explore more complex research questions.

## Introduction

Early specialization is a contentious topic and researchers continue to debate the optimal developmental pathway toward athletic expertise (e.g., Côté, 1999; Storm et al., 2012). Organizations including the American Medical Society for Sports Medicine (DiFiori et al., 2014), and the International Society of Sport Psychology (Côté et al., 2009), recommend against early specialization due to the potential physical and psychological risks. Recently, however, researchers have questioned the evidence base underpinning recommendations against early specialization (DiSanti and Erickson, 2019; Larson et al., 2019). This direction for inquiry possibly derives from the conceptualization of early specialization, and whether the measurement approaches are true to the generally accepted definition. Our overall aim is, therefore, to explore a new continuous measurement index that is better aligned to the general definition of early specialization. To address this aim, we will (1) illustrate the new index approach with a sample of aesthetic performers, and (2) critically evaluate the merits of the proposed index approach. We hope that exploring a new measurement approach will equip future researchers with the tools to examine more complex research questions, in relation to degrees of early specialization.

## Defining Early Specialization

A recent definition of early specialization is intensive participation in one sport, to the exclusion of others that is trained for and/or competed in for more than 8 months of the year, usually referring to children under ∼12 years^1^ (LaPrade et al., 2016). Interestingly, a recent review by DiSanti and Erickson (2019) found that a large percentage of articles exploring early specialization (67.5%) failed to cite a clear definition. Disparity between the conceptualization of early specialization is evident throughout the existing literature, perhaps due to the absence of definition-driven measurement approaches.

One approach to capturing early specialization is to measure just one component of the definition. For example, DiStefano et al. (2018) focused on whether athletes were single-sport or multi-sport, whereas others have used early high performance as an indicator of early specialization (e.g., Román et al., 2018). Bell et al. (2016) found that different methods for capturing specialization produced different results for reported prevalence. More recently, it has been suggested that studies which fail to capture training volume are missing a key marker of early specialization (Larson et al., 2019). Such inconsistencies in measurement approaches make interpretation of results, and comparison between studies, problematic.

When extensive training history data has been collected, the individual markers of early specialization have typically been analyzed separately. For example, Leite et al. (2009) collected data on general sport start age, main sport start age, length of training season, sampled activities, types of sports and weekly training volume, but these components were analyzed as individual predictor variables. Overall, existing literature fails to capture the accumulative nature of early specialization. Indeed, we believe that an early start age is not a significant marker of early specialization unless is it combined with year-round high-volume training in a single sport. We, therefore, argue that capturing the accumulative nature of early specialization is of key importance.

There is also disparity about whether early specialization is conceptualized as a continuum or as a dichotomous (yes/no) variable. If specialization is viewed as a process, whereby markers of early specialization can occur at different ages, a continuum approach would be most appropriate. Yet, DiSanti and Erickson (2019) found that over 90% of literature viewed specialization as dichotomous. One study concluded that defining athletes as specialized or non-specialized was insufficient when trying to differentiate the nuances between athlete experiences (Voigt and Hohmann, 2016). This is supported by reports that some athletes have a “main sport” from a young age but invest in their training much later (Storm et al., 2012). Therefore, methods that attempt to capture the complexity and accumulative nature of early specialization should be developed further.

As highlighted above, many factors make measurement of early specialization complicated, and the use of over-simplified measurement approaches should be addressed. In response, the aim is to explore a new continuous measurement index that is more able to deal with the complexities of early specialization, whilst remaining true to the general definition.

## Methodological Considerations for Measurement Development

To produce an index approach with adequate face validity, we believe that it is important to capture the four key aspects of the most well-used definition of early specialization including *training intensity*, *year-round training*, *single-sport* engagement, and all this taking place “*early*” (i.e., age 12 or younger; LaPrade et al., 2016). Below, we discuss how previous research has captured the four aspects of early specialization, and how we propose advancement of these measures (for the full questionnaire, see Supplementary Appendix).

### Training Intensity

According to LaPrade et al. (2016), for an athlete to be considered specialized the training intensity should be greater than that of a recreational participant. Generally, organized sport where there is a focus on skill acquisition is considered specialized, in comparison to more playful or unorganized forms of participation (Côté et al., 2009). Involvement in competitions may indicate the intense training that is associated with specialized athletes. Previous research has also used participation in an academy or talent development program as an indicator of specialized training (e.g., Clarke et al., 2018). A widely used indicator of training intensity is training volume (e.g., Baker, 2003; Bruce et al., 2013), which is typically calculated through retrospective interviews or questionnaires. As noted above, Larson et al. (2019) highlighted the importance of capturing training volume as an integral marker of early specialization.

In line with this previous research, we use training volume as the main marker of training intensity, whereby higher training volume indicates a higher degree of specialization. Average weekly training volumes are captured in three age categories (0–6 years, 7–9 years and 10–12 years), which is in line with previous research (e.g., Côté et al., 2007). These age categories are informed by the Developmental Model of Sport Participation proposed by Côté et al. (2007), which recommends that up to around age six is a child’s entry into sport and age 7–12 is considered the sampling years. Therefore, the 0–6 year age category is synonymous with this model, and the 7–9 years and 10–12 years categories are devised as equal divisions of the sampling years. Furthermore, recalling training history in age categories reduces the time needed to complete the questionnaire, in comparison to recalling training history for each year. Within these categories, participants report the typical length of a training session and the average amount of training sessions per week. These are multiplied to produce an average weekly training volume within each age category.

### Year-Round Training

Originating in Hill’s (1987) definition of specialized training, the concept of year-round training is essential. In 2015, Jayanthi et al. (2015) introduced the notion that training for more than 8 months of the year could capture year-round participation. Eight months as a cut-off value for year-round training was then included in LaPrade et al.’s (2016) updated definition of early specialization. However, the “>8 month rule” is not well-grounded in previous research and is therefore open to critique. Notably, clubs that follow a typical school calendar, which ranges from 34 to 38 weeks per year in European schools (Eacea, 2018), can generally be considered year-round by LaPrade et al. (2016) definition. Consequently, the majority of organized sports may be categorized as year-round, which questions whether specialized training being more intense than recreational engagement is truly captured by this “>8 month rule.” Indeed, a parent in a recent study commented on how there is pressure for young athletes to attend additional training during holidays to avoid being surpassed by peers (Patel and Jayanthi, 2018).

To measure year-round training, the participants report whether they trained during term time only (∼8 months/year), or more (term time plus some/most/all holidays), within the respective age categories. This wording is selected as organized activities typically follow the school calendar, which may be easier for athletes to recall than weeks or months spent in training. Having these gradations of year-round training beyond more/less than 8 months, allows differentiation of being specialized to a higher/lower degree.

### Single Sport

Another central marker of specialization is single-sport engagement. Early specialization generally refers to athletes who focus on one sport from a young age, and limit participation in other sports (Côté et al., 2007). Some researchers asked participants to list the number of sports they sampled during childhood (e.g., Law et al., 2007), with fewer sports indicating a greater degree of specialization. Unfortunately this, too, is problematic, as there is no clear definition of what multi-sport (or sampling) participation is. For example, one study highlighted that, at least from a Danish perspective, sampling sports is ingrained in school physical education so it is not possible for an athlete to have exposure to just one activity (Storm et al., 2012). We therefore believe that, until a clearer definition of sampling is devised, listing participation in other physical activities is the best available marker for single sport participation. Additional information, such as participants self-reporting when they felt they were prioritizing training within one activity, is also recommended.

To capture single activity engagement, participants report at which age they began to make sacrifices in favor of their main activity (e.g., dropping out of other sports). Making sacrifices early is then seen as indicating a greater degree of early specialization. To complement this question, athletes also list up to six additional physical activities that they have participated in for at least one school term, including ages at which they started and, where applicable, stopped. Only activities that were sampled up to, and including, age 12 are included in the analysis. This procedure makes it possible to get an indication of how specialized an athlete’s journey has been, as sampling fewer sports is considered a higher degree of early specialization. However, due to the overall weakness in the conception of single sport participation and sampling, this marker has a lower overall weighting in the calculation for degrees of specialization.

### Specializing “Early”

If an athlete participates in intensive, year-round training in a single sport ≤12 years, they would be considered an early specializer (Côté et al., 2007; LaPrade et al., 2016). The exact cut-off age for when specialization should be considered early is inconsistent throughout previous literature, however (DiSanti and Erickson, 2019). There are several studies that focused on age-based developmental milestones, such as age of starting competitions or being selected into a talent development program, to capture the age at which specialization occurred (e.g., Hayman et al., 2011; Bruce et al., 2013). However, sports have different norms in terms of start age. For instance, such that what constitutes “early” in one activity (e.g., specializing at age 23 in marathon; Noble and Chapman, 2018) may be considered late in another (e.g., gymnastics; Kerr et al., 2015).

To address the contentious issue around the “cut-off” for when specialization should be considered early, we propose utilization of a continuous approach. For example, specializing at, say, age 9 is earlier than age 11, as opposed to early simply referring to specialization ≤ age 12. Specifically, participants self-report what ages they (a) began training in their main activity, (b) began training with a focus on performance or competition, (c) were selected into an exclusive training program and (d) began making a serious investment in their training. We consider those who report reaching these milestones at earlier ages to have specialized early to a higher degree. Those who have not reached a milestone by 13 years old are considered non-specialized in relation to that specific marker.

## Illustration of Index Approach

Below we illustrate the index approach, which aims to capture early specialization by focusing on the four key components of the definition (training intensity, year-round training, single sport, commencing ≤12 years), within a sample of aesthetic performers. We also explore the application of the index scoring system and the overall suitability of the measure.

For the purpose of this study, aesthetic activities are defined as activities where pre-pubertal success is typical, performance is judged or valued subjectively, and is characterized by attire which reveals bodily contour and enhances artistic qualities. As such, participants were recruited from aesthetic sports and dance. Although not considered a sport, we argue that dance is a highly selective activity that mirrors the demands of sports. Competitions, such as the Dance Grand Prix and Prix De Lausanne, are also becoming increasingly common.

### Participants

There were 290 aesthetic performers aged 12–20 years (*M* = 15.88 years old, *SD* = 2.34; 83% female) recruited for the study. This age range was selected as the higher age limit allowed us to recruit from national teams and pre-professional dance schools. The lower age limit was chosen as the index is calculated from training history data up to, and including, age 12. Participants were recruited from suitable schools, clubs and teams within Sweden. The sample comprised 115 gymnasts (25 artistic; 22 rhythmic; 51 team; 17 trampoline), 71 dancers (including a variety of styles, such as ballet, jazz and modern), 69 figure skaters, 27 divers and 8 synchronized swimmers. The performers trained an average of 8.30 times per week (*SD* = 5.43) and >70% were training at national or international level (sport), or were in vocational or professional training (dance).

### Procedure

Before starting the study, the Swedish Ethics Review Authority granted ethical approval in line with national, European and international guidelines. Appropriate schools, clubs and national teams in Sweden were emailed information about the study, and interested coaches/teachers were then asked to distribute information to performers. Parental consent was obtained where required and informed consent was obtained from all participants. Pilot tests were conducted with three groups of dancers and gymnasts aged 12–16 years (*n* = 32; *M* age = 14.72). These pilot tests provided an estimate for how long the questionnaire takes to complete, as well as verifying participants’ comprehension of the questions. Questionnaires were administered at training venues with a researcher present (*n* = 240), or sent to the performers home address and returned via pre-paid post (*n* = 50). No incentive was given for participation, however a small snack was provided for participants completing the questionnaire at their training venue, where this was wanted.

### Workshops

In line with the key markers of early specialization, we created an index approach that aims to capture degrees of early specialization. As not all the data collected in the questionnaire is continuous, a weighting system is required for each individual component of the questionnaire. The weighted scores are added together to create a final index score (see Table 1). A high score indicates that the performer specialized early to a high degree, and a score of or near zero indicates that the performer was not an early specializer.

**TABLE 1:** Scoring for early specialization index.

	0–6 years	7–9 years	10–12 years	≥13 years
**Intensity**				
Hours per week	0 = 0	0 = 0	0 = 0	N/A
	<1 h = 1	<2 h = 1	<4 h = 1	
	1–2 h = 2	2–4 h = 2	4–8 h = 2	
	2–3 h = 3	4–6 h = 3	8–12 h = 3	
	>3 h = 4	>6 h = 4	>12 h = 4	

**Year-round**				
Time in training	Term time (∼8 months per year) = 1			N/A
	Term time plus occasional holidays = 2			
	Term time plus most holidays = 3			
	Term time plus all holidays = 4			

**Single sport**				
Number of other activities	0 = 3			N/A
	1 = 2			
	2 = 1			
	>3 = 0			
Age when significant sacrifices were made for main activity	3	2	1	0

**Specializing “early”**				
Age when started main activity	3	2	1	0
Age when became highly dedicated to main activity	3	2	1	0
Age of selection into group	3	2	1	0
Age when began training specifically for performance and/or competition	3	2	1	0

**Example scoring: A performer who reports training for 1.5 h before age 6 (2 points), 3 h at ages 7–9 (2 points), 9 h at ages 10–12 (3 points), this training is term time only before age 6 (1 point) and at ages 7–9 (1 point), at age 10–12 the athlete trained term time plus some holidays (2 points), more than three activities were sampled before age 12 (0 points), they began training at age 4 (3 points), dedicated at age 11 (1 point), were selected at age 12 (1 point), began training specifically for performance and/or competition at age 9 (2 points) and made sacrifices in favor of their sport at age 9 (2 points), would score a total specialization index of 20 out of a possible 42 points.**

To ensure the scoring system was functional and produced a suitable range of values, expert consultation workshops were conducted. Specialists from aesthetic sports (*n* = 3), dance (*n* = 3), and an academic whose research focuses on relevant theoretical frameworks (*n* = 1) attended these workshops to give their expertise on early specialization pathways within aesthetic activities. The attendees represented high performance coaches, teachers at top institutions, ex-professional performers and federation officials, who were deemed to be deeply knowledgeable within their field. The aim of these workshops was to discuss each question in relation to what would be considered high, and low, degrees of specialization. For example, within a particular age category weekly training volume should be scored between zero (no specialization) and four (high specialization), but ideally not with a large proportion of participants scoring zero (floor effect) or four (ceiling effect). The first author introduced the theoretical underpinning of the index approach before opening up a discussion on each of the index components. Several relevant themes were raised. For example, prior to the workshops, typical weekly training volume had been underestimated. Specifically, we (the authors) posited that >10 h of training would be considered “a lot” (index weight of 4) for an performer aged 10–12 years; however, during the workshops the experts expressed that this estimate was low, and it was therefore altered (see Table 1 for details of the index weights).

## Results

Here we present our descriptive results to illustrate how the index approach functioned with our sample of aesthetic performers. Table 2 highlights that early specializers were typical within the sample, as large percentages of the participants reported reaching specialization markers ≤12 years old. There are no guidelines on what training volume would indicate specialization; however, in the present sample, training hours increased relatively sharply from just over 1 h per week in the 0–6 age category, and up to just over 8 h per week in the 10–12 age category.

**TABLE 2:** Descriptive statistics for early specialization index.

		All participants		For those who were training		
	≤12 years	*M*	*SD*		*M*	*SD*

Age when		Years				
Starting main activity	94.5%	6.88	3.03			
Selected	74.4%	10.22	3.04			
Invested	71.1%	11.43	2.50			
Began focusing on performance/competition	82.5%	10.18	2.53			
Sacrifices were made in favor of main activity	72.4%	11.31	2.52			

**Average weekly training volume**		**Hours**		**Hours**		

0–6 years		1.29	1.94	*n* = 149	2.39	2.09
7–9 years		3.74	3.49	*n* = 217	4.75	3.26
10–12 years		8.07	5.34	*n* = 263	8.66	5.05

Year-round training*						

0–6 years		0.75	0.90	*n* = 140	1.44	0.74
7–9 years		1.60	1.23	*n* = 209	2.06	1.01
10–12 years		2.42	1.15	*n* = 254	2.60	0.97
Number of sampled activities ≤12 years		2.24	1.45			

*Year-round training is collected on an ordinal scale from 0 (not training) to 4 (training during term time and all holidays). The column “≤12 years” represents the percentage of participants who reached each specialization milestone at age 12 or younger. The column “for those who were training” shows the descriptive statistics when those who had not begun training in that particular age category had been removed.

Some variables are skewed as a result of late starters within the sample, and therefore Spearman’s rank order was used for correlational analyses (see Table 3). Generally, moderate to high correlations were evident between the variables, with the exception of sampling activities which only correlated with training volume; perhaps those who trained more had less time to sample other activities. Expectedly, weekly training volume and year-round training in the 0–6 years age group correlated very highly. Both are nevertheless included in the index calculation, because training intensity and year-round training are separate components of the early specialization definition used (LaPrade et al., 2016). Additionally, correlations between weekly training volume and year-round training in the other age categories were more moderate (*r* = 0.26–0.79).

**TABLE 3:** Bivariate correlations between index variables.

		1	2	3	4	5	6	7	8	9	10	11	12
**Age when**													
1	Starting main activity												
2	Selected	0.49**											
3	Invested	0.39**	0.63**										
4	Began focusing on performance/competition	0.51**	0.74**	0.75**									
5	Sacrifices were made in favor of main activity	0.39**	0.49**	0.64**	0.58**								
**Average weekly training volume**													
6	0–6 years	−0.77**	−0.47**	−0.32**	−0.50**	−0.27**							
7	7–9 years	−0.61**	−0.62**	−0.57**	−0.69**	−0.48**	0.64**						
8	10–12 years	−0.40**	−0.50**	−0.56**	−0.64**	−0.49**	0.48**	0.71**					
**Year-round training**													
9	0–6 years	−0.76**	−0.49**	−0.32**	−0.49**	−0.30**	0.92**	0.57**	0.40**				
10	7–9 years	−0.53**	−0.64**	−0.52**	−0.64**	−0.53**	0.48**	0.79**	0.56**	0.52**			
11	10–12 years	−0.25**	−0.51**	−0.49**	−0.58**	−0.48**	0.26**	0.51**	0.55**	0.32**	0.67**		
12	Number of sampled activities ≤12 years	0.09	0.07	–0.04	0.08	0.01	−0.16**	−0.10*	−0.10*	−0.16**	0.01	0.04	
13	Specialization Index	−0.69**	−0.77**	−0.69**	−0.84**	−0.64**	0.73**	0.88**	0.77**	0.72**	0.83**	0.65**	−0.18**

**Correlations on variables 1–12 are calculated from raw data.**

### Index Scoring and Calculation

Participants were prompted to report their start age for their main activity, yet some participants (*n* = 29) reported sampling children’s dance/gymnastics before this age. For those who reported training volume for their prior participation in children’s gymnastics/dance (*n* = 5), the start age was lowered to align with the training history data. This decision reflects that those who reported training history likely felt this was an important part of their development in their main activity. The start age remained unchanged for those who reported sampling children’s dance/gymnastics but did not provide training volume for this (*n* = 24).

As a result of missing data on one or more markers, 247 participants, out of a possible 290, had adequate data to calculate an index score. There were two potential ways in which data could be missing: (1) occasional omissions, where the participant intentionally or unintentionally did not answer the question, and (2) data missing because the participant was not training during a particular age band. The latter cause of missing data was typically dealt with during data collections, as participants were asked to write zero for times when they were not training. However, in cases where this information was missing, it was possible to refer back to the reported start age to gauge whether or not the participant would, or would not, have been training during a specific age category. After dealing with missing data in relation to training volume, Little’s MCAR test was non-significant (*p* = 0.50) for all index markers; therefore there was no systematic patterns to the missing data.

Despite the adaptations made to the index through the expert consultation workshops, ceiling effects existed in relation to weekly training volume, when index weightings were applied to the data. As the aim was to create an index capable of distinguishing degrees of early specialization between participants, it was important that not too many yielded the top score for any particular index marker. Although adjustments were therefore made to the scoring system, the suggested weightings were largely agreed upon by experts and researchers, and remained functional throughout the analysis phase. Table 4 shows how the index scoring was adapted, in order to avoid ceiling- or floor effects. As a result of data driven adjustments, the composite index variable was normally distributed with a range of 0–36. The complete index scoring system can be seen in Table 1.

**TABLE 4:** Index frequencies for average weekly training volume (10–12 years).

Score given	0	1	2	3	4
Theorized training volumes	0	≤180	181-360	361-600	>601
Frequency (*n*)	19	26	89	68	80
Data driven training volumes	0	≤240	241-480	481-720	>721
Frequency (*n*)	19	37	108	71	47

**Training volume is given in minutes.**

## Critical Evaluation

We will move on to our second aim and critically discuss the conceptualization and usage of the proposed early specialization index, and offer suggestions for future research. Specifically we deliberate the barriers to creating a definition-driven index, including the ambiguity of several key components. These critical evaluations are provided under two subheadings: Questionnaire and scoring evaluations, and Analytical evaluations.

### Questionnaire and Scoring Evaluations

Future researchers may wish to discuss the ambiguity of what should, and should not, be included as training. Previous researchers (Côté et al., 2007) outlined that early specialization is characterized by deliberate practice (Ericsson et al., 1993); that is, training that is focused on skills acquisition rather than enjoyment. However, exactly what training types qualify as deliberate practice, and whether athletes are able to recall this retrospectively, is widely disputed (e.g., Baker and Young, 2014). To manage the issue of what “counts,” participants in the present study were asked to include training relating to their main activity that was either coach or self-led, and that focused on developing or practicing skills. This was deemed most suitable due to the mix of sports and dance styles within our sample. Other researchers, who may sample within a single sport or activity, would be able to further define what type of activities should be included in training volume calculations.

An additional point of contention is how an individual index marker is weighted. For the proposed index calculation, most questions were scored on a scale of 0–3, but training volume questions were scored on a scale of 0–4. This is justified as training volume is a pivotal component of early specialization and may require further distinction. In total there are 12 points available for three of the four components (training intensity, year-round training, and involvement before age 12) and six points for one component (single sport). Single sport has the lowest overall weighting because of the general weakness surrounding the definition of sampling.

When designing the index approach we were required to decide how many sampled activities might be considered “a little” or “a lot”; however, no current guideline exists for this. There is a general notion that sampling is positive for long-term athlete development (Côté et al., 2007; Côté and Erickson, 2015). It is surely not the case, however, that more is always better. It must be possible to participate excessively in sport, perhaps risking overtraining even if far from specialization. Therefore the data-driven approach was essential to inform what was considered a high level of reported physical activities within the sampled participants. As such, reporting three or more activities ≤ age 12 scored zero points (i.e., not specialized). As researchers further develop the definition of sampling, the way in which it is captured as a marker of early specialization should be adapted. Until then, we believe using the data-driven approach outlined in this paper is the most suitable, as it provides an overview of what is a high and low level of sampling.

Finally, requesting participants’ start age in their main activity was unexpectedly complex. Many activities have a general introductory stage (e.g., children’s general gymnastics, mixed children’s dance classes) before they commence in, for instance, a particular form of gymnastics (e.g., artistic) or dance style (e.g., jazz). This raises questions about what activities are prerequisites for early specialization. For example, should participation in mixed children’s dance/movement classes be considered the beginning of a specialized journey in both ballet and contemporary dance? In previous research into early specialization there is often little, or no, information concerning what signifies a young performer’s entry into their main activity. As such, future researchers may wish to further investigate how early specializers are introduced to their main activity.

### Analytical Evaluations

The expert consultation workshops functioned, amongst other things, as a way to theorize how an “average participant” would respond to the questionnaire. These workshops aided in drafting the original index calculation, however data driven adjustments were still required to avoid ceiling or floor effects. It is likely that more, and larger, changes may have been needed if the workshop had not taken place. We also acknowledge that future researchers may wish to implement this measurement approach outside of aesthetic activities. Although the index weightings were created specifically for aesthetic activities, we recommend future researchers to use the calculation outlined in Table 1. However, if the data is significantly skewed, or ceiling/floor effects are observed, and between study comparison are not essential, expert consultation workshops could be conducted to inform adjustments the index weightings. We anticipate that the index is most suitable for use in activities where early specialization is commonplace, and may be less appropriate for use in sports where specialization typically occurs later. For example, athletes may specialize earlier in individual than in team sports (Fahlström et al., 2015), which may be reflected in the possibility to have high training volume from a young age in individual sports. Making study-specific adjustments is beneficial for certain research questions as it may provide results that are more meaningful and increase content validity within a given population.

To increase the generalizability of this measurement tool, we recruited participants from various aesthetic activities. As such, we tested our measurement tool within aesthetic activities, including gymnastics, figure skating, synchronized swimming, diving and dance. Although not the aim of this study, future researchers may wish to sample within just one activity, or conduct between-group analyses to check for homogeneity.

The 12–20 age range utilized for this study may give rise to memory/recall biases, however no participants expressed such difficulties during data collections. Furthermore, previous retrospective-based research has been conducted in a variety of age ranges. Therefore, this limitation is not unique to the present study. As this index has been tested with performers aged 12–20, we suggest further researcher remain within this range. However, it is possible that age may affect interpretation some of the questions. Specifically, the age at which a performer recalls making significant sacrifices in favor of training may change over time. For example, what may appear to be a large sacrifice to a 12 year old, is likely different to that of a 20 year old. Furthermore, the younger participants in the study may go on to drop out of training before they reach 20 years, whereas the older participants represent those who have continued in their training. Therefore, researchers may wish to narrow this age range in future studies.

## Conclusion

Better aligning measurement of early specialization with its definition was a key motive for designing a new measurement approach. As such, we have outlined and explored an index approach for capturing degrees of early specialization. We believe this is a step toward improving overall understanding of early specialization. By aligning to the current definition, we are able to draw our conclusions with a degree of confidence in the internal validity. Indeed, creating an index to capture degrees of early specialization is also the first step in approaching research questions in a more nuanced way. For example, exploring the relationship between athletes’ degree of early specialization and postulated outcomes such as dropout, reduced enjoyment and injury (Côté et al., 2009) would be possible. Consequently, we urge future researchers to reconsider the use of dichotomous measures and single variable analysis of early specialization, in favor of an index approach.

Progressing measurement approaches heavily relies on a solid definition of early specialization. Particularly, researchers should address the definition of sampling and to what extent it impacts on athletes’ specialization. As revisions are made to the definition, measurement approaches will need to be developed further to remain relevant. For now, however, we believe the measurement index explored in the present study is a significant advancement on the previously used methods for capturing early specialization.

## Data Availability Statement

The datasets generated for this study are available on request to the corresponding author.

## Ethics Statement

The studies involving human participants were reviewed and approved by Swedish Ethical Review Authority. Written informed consent to participate in this study was provided by the participants’ legal guardian/next of kin.

## Author Contributions

All authors listed have made a substantial, direct and intellectual contribution to the work, and approved it for publication.

## Conflict of Interest

The authors declare that the research was conducted in the absence of any commercial or financial relationships that could be construed as a potential conflict of interest.

## Footnotes

1 Other researchers have previously provided definitions of early specialization (for example, Hill, 1987; Wiersma, 2000). However, both definitions are captured by LaPrade et al. (2016) definition.
