# Supplementary material for: An Index Approach to Early Specialization Measurement: An Exploratory Study
Source: Front Psychol. 2020 May 26;11:999. doi: 10.3389/fpsyg.2020.00999 (PMC7265620; doi:10.3389/fpsyg.2020.00999)
Supplement: Supplementary file 1 [file Data_Sheet_1.PDF]

Appendix 1 Early specialization index questionnaire

What is your main activity? _____________

How old were you when you:

- Started training in your main activity?
  - Age _____

- Were selected into an elite team, group, or training program?
  - Age _____ I have not been ☐

- Started training specifically for the purpose of performance/competition?
  - Age _____ I have not ☐

- Became highly dedicated to your training with the intention of high level performance?
  - Age _____ I have not ☐

- Made sacrifices (e.g. stopped other training/hobbies) in favour of your main activity?
  - Age _____ I have not ☐

Please enter how much, on average, you trained during the following age categories:

- When I was 6 years and younger I trained…
  - ____ times per week
  - ____minutes per session

  ☐ Term time only
  ☐ Term time plus some holidays
  ☐ Term time plus most holidays
  ☐ Term time plus all holidays.

This is repeated for each of the following age groups: 7-9 years, 10-12 years

Which other physical activities have you participated in?

- Activity 1: ____________
  - Age when you started? ______
  - Age when you stopped? ______

  ☐ I still do this activity

This is repeated with room for up to 6 activities. Participants to mark a dash if this is left intentionally blank.
